# The effects of acute aerobic activity on cognition and cross-domain transfer to eating behavior

**DOI:** 10.3389/fnhum.2014.00267

**Published:** 2014-04-30

**Authors:** Cassandra J. Lowe, Peter A. Hall, Corita M. Vincent, Kimberley Luu

**Affiliations:** ^1^School of Public Health and Health Systems, University of WaterlooWaterloo, ON, Canada; ^2^Department of Kinesiology, University of WaterlooWaterloo, ON, Canada

**Keywords:** aerobic exercise, acute exercise, executive function, snack foods, food intake

## Abstract

Prior studies have demonstrated that a single session of aerobic exercise can enhance cognitive functioning; specifically, the inhibition facet of executive function (EF). Additionally, previous research has demonstrated that inhibitory abilities are essential for effective dietary self-control. However, it is currently unknown whether exercise induced enhancements in EF also facilitate self-control in the dietary domain. The present study sought to determine whether a single session of aerobic exercise enhances EF, and whether there is a transfer effect to dietary self-control. Thirty four undergraduate students were randomly assigned to one of three exercise conditions: (1) minimal exercise; (2) moderate intensity exercise (30% heart rate reserve); (3) vigorous intensity exercise (50% heart rate reserve). After the exercise bout, participants completed three standardized EF tasks followed by a bogus taste test for three appetitive snack foods (milk chocolate and potato chips) and two control foods (dark chocolate and crackers). The amount of food consumed during the taste test was covertly measured. The results revealed a significant main effect of treatment condition on the Stroop task performance, but not Go-NoGo (GNG) and Stop Signal task performance. Findings with respect to food consumption revealed that EF moderated the treatment effect, such that those with larger exercise effects on Stroop performance in the moderate intensity exercise condition consumed more control foods (but not less appetitive foods). These findings support the contention that a single bout of aerobic exercise enhances EF, and may have transfer effects to the dietary domain, but that such effects may be indirect in nature.

## Introduction

There has been recent interest in the beneficial effects of acute bouts of aerobic exercise on cognition. A recent meta-analysis suggested that acute bouts of aerobic activity are associated with a small but reliable positive effect on cognitive performance (*ES* = 0.20; Lambourne and Tomporowski, 2010). Moderators of the effect size include temporal sequencing of cognitive assessment in relation to exercise (following bout>during bout), modality of aerobic training (cycling>running), and task type (memory>processing speed; Lambourne and Tomporowski, 2010). With respect to executive function (EF) in particular, results have been variable with some studies showing moderate-to-large effects (Chang and Etnier, 2009; Pontifex et al., 2009; Chang et al., 2011), and others showing no beneficial effect (Tomporowski and Ganio, 2006; Coles and Tomporowski, 2008). These inconsistencies may be related to study design, exercise type, or facet of EF examined. To date, the majority of studies examining the effects of acute aerobic exercise on EF have focused on the inhibition facet of EF using the Stroop task (Hogervorst et al., 1996; Sibley et al., 2006; Barella et al., 2010; Lambourne and Tomporowski, 2010; Yanagisawa et al., 2010). Of these, Stroop task performance tended to improve following exercise in both young (Hogervorst et al., 1996; Sibley et al., 2006; Yanagisawa et al., 2010) and older adults (Barella et al., 2010). Furthermore, Yanagisawa et al. (2010) demonstrated that acute aerobic exercise increased cortical activation of the left dorsolateral prefrontal cortex (DLPFC) during the Stroop task, and this enhanced activation corresponded with improved performance on the Stroop task. Taken together, these studies suggest that acute aerobic exercise especially enhances the inhibitory control facet of EF.

Nonetheless, it remains unclear what, if any, implications exercise-induced enhancements in inhibitory control might have on everyday activities that rely on such abilities. Recent studies have suggested that inhibitory abilities may play an important role in self-regulatory processes required for dietary self-restraint (Rotenberg et al., 2005; Guerrieri et al., 2009, 2012; Nederkoorn et al., 2009; Houben and Jansen, 2011; Hall, 2012; Hall et al., 2013). If enhancements in EF—and inhibition specifically—are induced by acute aerobic activity, it is possible that such enhancements may facilitate self-control in the dietary domain as well, either directly or indirectly. Exercise induced enhancements in EF may directly facilitate dietary self-control by moderating the quantity of food consumed. However, it is also plausible that such enhancements may indirectly facilitate dietary self-control, by moderating the type of food consumed (i.e., healthy vs. unhealthy foods). The current study was designed to test these possibilities. It was hypothesized that aerobic activity would enhance EF, particularly when measured with the Stroop task, and that improvements in Stroop performance would predict reduced consumption of appetitive (unhealthy) foods in a subsequent laboratory taste test paradigm. We also examined the possibility of indirect transfer to consumption of control (perceived healthier) foods.

## Methods

### Participants

Thirty-four undergraduate students, aged 18–27 (*M* = 20.24; *SD* = 1.76), were recruited from psychology courses; sample characteristics are presented in Table 1. Participants received course credits in exchange for their participation. All participants indicated that they were inactive (i.e., sedentary), and liked the experimental foods (milk chocolate and potato chips) in a pre-screening questionnaire; the screening procedure is outlined below. Written and informed consent was obtained from all participants. This study was reviewed by and received approval from the University of Waterloo Research Ethics Board, and was conducted in accordance with standard ethical protocols.

**Table 1:** **Participant demographic, EF task performance, and food consumption by exercise condition**.

	**Minimal (*n* = 12)**		**Moderate (*n* = 10)**		**Vigorous (*n* = 12)**	
	**Mean (*SD*)**	**% (*n*)**	**Mean (*SD*)**	**% (*n*)**	**Mean (*SD*)**	**% (*n*)**
Age (years)	19.5 (2.53)		19.5 (1.08)		20.17 (0.94)	
BMI	22.8 (3.7)		22.03 (2.64)		22.25 (3.36)	
Hunger	6.8 (2.61)		5.4 (1.51)		6.08 (1.8)	
**GENDER**						
Male		41.7 (5)		40.0 (4)		16.7 (2)
Female		58.3 (7)		60.0 (6)		83.3 (10)
**ETHNICITY**						
Caucasian/white		50.0 (6)		20.0 (2)		33.3 (4)
Asian		41.7 (5)		40.0 (4)		50.0 (6)
Black						8.3 (1)
Hispanic				10.0 (1)		
South Asian		8.3 (1)		10.0 (1)		8.3 (1)
Middle Eastern				20.0 (2)		
**EXECUTIVE FUNCTION MEASURES**						
GNG RT (ms)	404.05 (33.73)		392.91 (26.44)		410.43 (28.69)	
SST accuracy (% incorrect on stop trials)	0.19 (0.16)		0.17 (0.13)		0.20 (0.17)	
Stroop interference (ms)	315.51 (328.13)		−110.7 (336.67)		60.61 (293.36)	
**FOOD CONSUMPTION MEASURES**						
Total food consumed (grams)	75.36 (23.37)		89.0 (30.88)		78.42 (28.05)	
Total appetitive foods consumed (grams)	59.36 (22.74)		69.2 (25.6)		59.75 (20.36)	
Total Control Food Consumed (grams)	16.0 (5.67)		19.8 (8.8)		18.67 (12.04)	

### Pre-screen measures

Several weeks prior to study participation, potential participants completed a pre-screening package, which included items to identify sedentary individuals that also liked the experimental foods. Self-reported exercise was measured using the Bogg Exercise Scale (Bogg et al., 2008). Participants that exercised less than two times per week, according to this scale, were deemed inactive and eligible to participate in the study. In addition, the following two items (adapted from Hill et al., 1991) were used to identify participants who liked potato chips and chocolate: (1) “how often do you experience cravings to eat potato chips/chocolate?” (response scale: 1 = “never”; 10 = “all the time”); (2) “how strong are these cravings you experience to eat potato chips/chocolate” (response scale: 1 = “extremely weak”; 10 = “extremely strong”); individuals who scored 7 or above on the response scale for both items and both experimental foods were deemed eligible to participate in the study.

### Procedure

Eligible participants were tested individually in a single laboratory session lasting approximately 2 h. All participants were required to abstain from eating or drinking any caffeinated beverages 3 h prior to their scheduled session, with compliance checked upon their arrival. All laboratory sessions were conducted at the same time of day (3:30–5:30 PM). At the start of each exercise session, participants completed the Physical Activity Readiness Questionnaire (PAR-Q). The PAR-Q is a seven item questionnaire designed to screen for any health conditions that could be exacerbated by exercise; no participants were excluded due to health conditions.

For the aerobic exercise bout, participants were randomly assigned to one of three exercise conditions: (1) minimal exercise; (2) moderate intensity exercise (30% heart rate reserve); (3) vigorous intensity exercise (50% heart rate reserve). Exercise bouts were conducted using a recumbent cycle ergometer. Participants were fitted with a heart rate monitor prior to exercise, and heart rate was monitored continuously and recorded every 5 min. Resting heart rate was measured after participants rested for 1 min the cycle ergometer prior to exercise.

For the minimal exercise condition, participants cycled at a slow and steady rate, 30–40 rpm, without significantly increasing their heart rate. The exercise session lasted a total of 35 min, and consisted of a 5 min warm-up, 25 min of exercise at target heart rate (THR) (the first 5 min were used to bring heart rate up to the THR), and a 5-min cool-down.

For the moderate exercise condition, participants began pedaling at 60–70 rpm. Work load was then increased in 10 W increments to gradually raise heart rate from resting to the THR. THR was established based on heart rate reserve (HRR). Heart rate reserve was calculated as maximal heart rate (MHR), estimated using the formula 220-age, minus resting heart rate (RHR). Next, THR was calculated by multiplying HHR by the target intensity, 30% for the moderate condition and 50% for vigorous condition, and adding it to RHR (THR = RHR + (MHR − RHR) TI%). Exercise duration was the same as that for minimal condition. The vigorous condition was identical to the moderate condition except that intensity was established at 50% HRR.

Immediately following the exercise bout, participants completed three computer-administered EF tasks; the order of the tasks was counterbalanced across participants. The total duration of all three EF tasks was approximately 30 min. Directly following the EF tasks, participants were asked to report their current subjective level of hunger (on a 1–10 scale), and subsequently completed a bogus taste test. Prior to each experimental session, the weight of the experimental foods were measured and recorded. For the taste test, participants were instructed to taste and rate the subjective properties (i.e., texture, sweetness, and saltiness) of each experimental food. Participants received instructions to consume as much food as they would like, and to tell the experimenter when they had completed their ratings. During the taste test, the experimenter left the room until the participant indicated they had completed the taste test, at which point the next food was presented; the time each participant took to complete their taste ratings was covertly measured. On average, the total duration of the taste was 13 min (*SD* = 5.58). The experimental foods were presented in the following order: (1) Belgian milk chocolate; (2) Belgian dark chocolate; (3) regular potato chips; (4) flavored potato chips; (5) soda crackers. Participants were not provided with any information regarding the macronutrient content of the experimental foods. Following the taste test, the experimental foods were weighed and the amount of food consumed (grams) during the taste test was recorded.

### Food consumption measures

The taste test foods were divided into two categories: (1) appetitive foods (milk chocolate and potato chips); (2) control foods (dark chocolate and crackers). The following item from the taste rating questionnaire was used to confirm that participants perceived the appetitive foods as more appealing than the control? foods “Overall, how would you rate this food?” (response scale: 1 = “not at all good”; 10 = “very good”). As expected, participants rated the appetitive foods as significantly more appealing than the control foods [*t*_(33)_ = 9.266, *p* < 0.001].

### Executive function measures

All EF measures were presented via E-Prime software (Psychology Software Tools, Inc) on a desktop computer; participant responses were made via button press using a response box. Participants were instructed to respond as quickly and accurately as possible for all tasks.

### Stroop task

The Stroop task (Stroop, 1992) was modeled after the variant in Miyake et al. (2000). The Stroop task is a reliable EF measure (Strauss et al., 2005; Friedman et al., 2008), and is one of the most widely used measures of response inhibition. In this particular version of the task, participants were instructed to name the color of stimulus presented on a computer screen. All stimuli were presented individually in one of six colors: blue, green, orange, purple, red, or yellow. The task consisted of a mixed block of trials containing 72 trials with a string of asterisks, 60 incongruent color word trials (e.g., the word blue appearing in red colored font) and 12 congruent color word trials (e.g., the word blue appearing in blue colored font). For each trial, the stimuli were presented on the screen until the participant responded, followed by a response to stimulus interval of 1000 ms minus the response time. The crucial dependent variable was the Stroop inference effect, calculated as the reaction time on correct incongruent trials minus the reaction time on correct congruent trials; shorter reaction times were taken to reflect stronger EFs.

### Go/No-Go task

The Go-NoGo (GNG) task is a widely used and reliable measure of response inhibition (Kuntsi et al., 2005). In this variant of the GNG task, participants were instructed to press a button as quickly as possible whenever a lower case letter was presented on the computer screen, and withhold their response whenever an upper case letter appeared on the computer screen. For each trial, the stimulus duration was set at 1000 ms, with a 500 ms interstimulus interval. The task consisted of eight blocks, with 60 trials in each block. In half of the test blocks upper case letters were predominant (5:1 ratio) and in the other half of the test blocks lower case letters were predominant (5:1 ratio). The crucial dependent variable was reaction times on correct trials; shorter reaction times were taken to be indicative of stronger EFs.

### Stop signal task

The Stop Signal Task (SST; Logan et al., 1984) was modeled after the variant in Miyake et al. (2000). Like the other EF tasks, the SST is a reliable measure of inhibition (Friedman et al., 2008; Congdon et al., 2012). In this particular version of the SST task, participants completed two blocks of trials. The first block of 48 trials was used to build up a prepotent categorization response. Participants were instructed to categorize a series of words, presented individually on a computer screen, as an animal or non-animal word. During the second block of 96 trials (stop trials), participants completed the same categorization task, but they were instructed to not respond (i.e., withhold their response) when they heard a computer emitted tone (stop signal; 23 trials). The stimulus duration was 1500 ms with a 500 ms interstimulus interval. The stop signal delay (the duration between the onset of trial and the time at which the stop signal occurred) was adjusted for each participant by subtracting 225 ms from the mean reaction time on go trials. The crucial dependent variable was the proportion of incorrect responses on stop trials (i.e., responding when the stop signal was present); higher accuracy on the stop trials was taken to signify stronger EFs.

### Statistical analytic procedure

The accuracy for the both the Stroop (*M* = 0.94; *SD* = 0.11) and the GNG (*M* = 0.89; *SD* = 0.14) tasks were uniformly high, and so analyses focussed on accuracy-corrected reaction times in both cases. To reduce the potential influence of outliers, a Winsorizing technique was applied to outlier reaction times for the Stroop and GNG task; outlier scores were removed and replaced with the next sequential value. Next, to confirm group randomization, a one-way Analysis of Variance (ANOVA) was conducted to compare age, sex, current hunger, and BMI across experimental conditions.

To test the hypothesis that a single session of aerobic exercise would enhance EFs, a one-way ANOVA was conducted to compare performance on the Stroop, GNG, and SST across exercise conditions. Significant main effects were followed up with planned Fisher's LSD comparisons. Following this, a one-way ANOVA was conducted to determine if there was a significant treatment effect on the total amount of food consumed, the total amount of appetitive food consumed, and the total amount of control food consumed. Next, hierarchical moderated multiple regression analyses were conducted to determine if there was a moderating effect of EF on food consumption across treatment conditions. Following the procedures outlined in Aiken and West (1991), effect coding was used to denote group membership; the experimental conditions (i.e., exercise condition) were coded into two vectors (i.e., +1, 0, −1). The main effect variables (exercise condition and EF scores) were centered and combined into an interaction term. The main effects were entered on the first step of the hierarchical linear regression analysis, followed by the interaction terms on the second step. Separate regression analyses were conducted for each EF task to determine if (1) there is a moderating effect of EF on the total amount food consumed, and (2) if there is a moderating effect of EF on the differential consumption of appetitive (milk chocolate and potato chips) and control (dark chocolate and crackers) foods. All statistical analyses were conducted using SPSS software (version 21; IBM Corp, 2012). All statistical analyses were determined a priori.

## Results

The three experimental groups did not differ significantly with respect to age [*F*_(2, 31)_ = 1.878, *p* = 0.170], sex [*F*_(2, 31)_ = 1.016, *p* = 0.374], BMI [*F*_(2, 31)_ = 0.18, 2, *p* = 0.835] and subjective hunger ratings [*F*_(2, 31)_ = 0.39, *p* = 0.683], indicating that randomization was successful. Zero order correlation for study variables are presented in Table 2. Across all participants, none of the variables were significantly correlated with the total amount of food, the total amount of appetitive food, and the total amount of control food consumed; however, GNG reaction time was marginally significantly correlated with the total amount of food consumed (*p* = 0.06) and the total amount of control food consumed (*p* = 0.07). Stronger GNG and SST performance predicted significantly lower subjective hunger ratings. Food variables were significantly correlated with one another.

**Table 2:** **Correlations between measures**.

	**1**	**2**	**3**	**4**	**5**	**6**	**7**	**8**	**9**	**10**
1. Age	1									
2. Sex	−0.06	1								
3. BMI	0.19	−0.10	1							
4. Hunger	0.34^*^	0.17	0.18	1						
5. SST Accuracy	0.20	−0.01	0.38^*^	−0.35^*^	1					
6. GNG RT	0.36^*^	0.28	−0.08	0.29^*^	0.17	1				
7. Stroop interference	0.10	0.23	0.08	−0.16	0.01	−0.005	1			
8. Total food consumed	0.06	−0.19	0.06	0.12	0.05	−0.27^†^	−0.21	1		
9. Total appetitive food consumed	−0.03	−0.20	−0.04	0.13	0.02	−0.22	−0.20	0.95^**^	1	
10. Total control food consumed	0.26	−0.13	0.28	0.02	0.05	−0.26^†^	−0.13	0.64^*^	0.36^*^	1

Spearman's rho was used to calculate sex based correlations.

† Correlation is significant at the p < 0.10 level (1-tailed);

* correlation is significant at the p < 0.05 level (1-tailed);

** correlation is significant at the p < 0.01 level (1-tailed).

### Effects of exercise on EF

**Figure 1:**
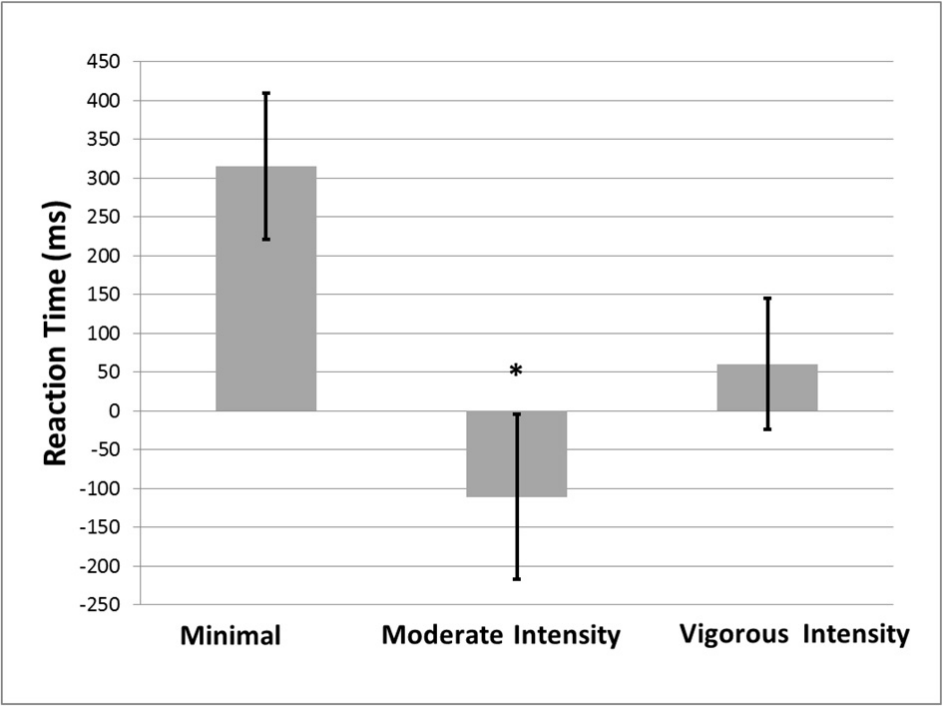
**Mean (±SE) Stroop interference effect (incongruent RT-congruent RT) as a function of exercise condition**. ^*^Significantly different from the minimal exercise condition at the *p* < 0.05 level (2-tailed).

A significant main effect of treatment condition on Stroop task performance [*F*_(2, 31)_ = 5.017, *p* = 0.013, *d* = −1.044] was observed. Compared to the minimal exercise condition, performance on the Stroop task was significantly better in the moderate intensity exercise condition (*p* = 0.004), and marginally better in the vigorous intensity exercise condition (*p* = 0.059; see Figure 1). No significant effects of treatment condition on GNG [*F*_(2, 31)_ = 0.942, *p* = 0.401, *d* = −0.053] or SST [*F*_(2, 31)_ = 0.122, *p* = 0.885, *d* = 0.011] performance were observed. The mean reaction times for GNG and Stroop, and the mean accuracy for SST by exercise condition are shown in Table 1.

### Effects of exercise on food consumption

There was no significant effect of experimental condition on the total amount of food consumed [*F*_(2, 31)_ = 0.703, *p* = 0.503, *d* = 0.282], the total amount of appetitive food consumed [*F*_(2, 31)_ = 0.622, *p* = 0.544, *d* = 0.204], or the total amount of control food consumed [*F*_(2, 31)_ = 0.466, *p* = 0.632, *d* = 0.332). That is, exercise did not differentially induce consumption differences across conditions for any food type or total food.

### Interaction between exercise and EF on snack food consumption

When total amount of food consumed was entered as the dependent variable, hierarchical moderated regression analyses indicated that there was no significant interaction between Stroop performance and the treatment effect for the vigorous (β = 0.173, *t* = 0.737, *p* = 0.467, *d* = 0.359) and moderate (β = −0.176, *t* = −0.747, *p* = 0.461, *d* = 0.368) intensity exercise group. Similar results were observed among the other two EF tasks: no significant interaction was observed between GNG performance and the treatment effect for the vigorous (β = −0.194, *t* = −0.898, *p* = 0.377, *d* = −0.404) and moderate (β = −0.070, *t* = −0.315, *p* = 0.755, *d* = −0.144) intensity exercise groups, and SST performance and the treatment effect for the vigorous (β = −0.086, *t* = 0.304, *p* = 0.763, *d* = −0.176) and moderate (β = 0.129, *t* = 0.395, *p* = 0.696, *d* = 0.268) intensity exercise groups. Comparable results were observed when examining the total amount of appetitive foods consumed. No interaction was observed between performance on the Stroop task and the treatment effect for the vigorous (β = 0.007, *t* = 0.030, *p* = 0.976, *d* = 0.014) and moderate (β = −0.007, *t* = 0.110, *p* = 0.913, *d* = −0.014) intensity exercise groups. Additionally, there was no interaction observed between GNG performance and the treatment effect for the vigorous (β = −0.078, *t* = −0.352, *p* = 0.728, *d* = −0.160) and moderate (β = −0.128, *t* = −0.561, *p* = 0.579, *d* = −0.265) intensity exercise groups, and SST performance and the treatment effect for the vigorous (β = 0.159, *t* = 0.564, *p* = 0.578, *d* = 0.329) and moderate (β = −0.032, *t* = 0.097, *p* = 0.924, *d* = −0.066) intensity exercise groups.

When examining the total amount of control foods consumed, a significant interaction between Stroop performance and the treatment effect was observed for the vigorous (β = 0.497, *t* = 2.285, *p* = 0.030, *d* = 1.179) and the moderate (β = −0.459, *t* = −2.100, *p* = 0.045, *d* = −1.069) intensity groups. Specifically, those in the moderate intensity condition showed a marginally significant positive association between Stroop performance and the amount of control food consumed (β = −0.617, *t* = −2.216, *p* = 0.058). There was no significant variability in the amount control food consumed in the vigorous (β = 0.380, *t* = 1.298, *p* = 0.224) and minimal (β = −0.158, *t* = −0.482, *p* = 0.642) intensity groups. A marginally significant interaction between GNG performance and the treatment effect was observed for the vigorous intensity exercise condition (β = −0.385, *t* = −1.819, *p* = 0.080, *d* = −0.856), such that those in the vigorous intensity exercise condition showed a marginally significant positive association between the amount of control food consumed and GNG performance (β = −0.513, *t* = −1.890, *p* = 0.088). There was no significant variability in the amount of control food consumed in the moderate intensity (β = −0.125, *t* = −0.355, *p* = 0.732) and minimal intensity (β = 0.145, *t* = 0.440, *p* = 0.670) groups. Furthermore, there was no significant interaction between SST performance and the treatment effect for the vigorous (β = −0.137, *t* = −0.486, *p* = 0.63, *d* = −0.283) and moderate (β = 0.305, *t* = 0.929, *p* = 0.361, *d* = 0.660) intensity exercise groups.

## Discussion

In the current study, we examined the effects of an acute bout of aerobic activity (moderate and vigorous) on cognitive function, and assessed transfer effects to a self-control task in the dietary domain. An acute bout of moderate aerobic activity significantly improved performance on the Stroop task. However, there were no significant improvements in Stroop task performance following vigorous aerobic exercise, or in GNG and SST performance for either intensity. The current findings of a significant effect of moderate aerobic exercise on Stroop performance is consistent with the findings of several prior studies (Hogervorst et al., 1996; Sibley et al., 2006; Barella et al., 2010; Yanagisawa et al., 2010). The null findings in relation to GNG and Stop signal suggest that the effects of aerobic activity are not uniform across all measures of EF, or even across all measures of the inhibition facet of EF. However, it is also possible that subtle aspects of the requirements unique to each of the specific tasks were differentially sensitive to exercise. Additionally, reliability differences between the tasks could also have affected results; it is possible that the null effects observed would be significant with a more well-powered experimental design (either within subjects or between subjects with a larger sample size).

With respect to food consumption, there was no significant difference in energy intake following an acute bout of moderate or vigorous aerobic exercise, a finding that is consistent with the results of several other studies (King et al., 1994, 1997a,b; George and Morganstein, 2003; Deighton et al., 2012). Contrary to the hypothesized result, there was no moderating effect of EF on consumption of appetitive foods. There was, however, a moderating effect of EF on consumption of control foods. Specifically, those with larger exercise effects on Stroop performance in the moderate exercise condition consumed more control foods compared to those in the vigorous and minimal exercise conditions. These results may be attributed to differences in the perceived healthiness of the control and appetitive foods. An accumulating body of evidence suggests that the consumption of dark chocolate, as opposed to milk chocolate, may reduce the risk of developing cardiovascular disease (Taubert et al., 2003; Engler and Engler, 2006; Erdman et al., 2008; Ried et al., 2010; Di Renzo et al., 2013), and therefore it is possible that the control foods were perceived as healthier than the appetitive foods. Consequently, given that participants reported being moderately hungry prior to the start of the taste test, it is plausible that those with larger exercise effects on EF consumed more control foods as a means of energy compensation while still exerting self-control (i.e., consuming more of the perceived healthier foods). This contention is further supported by the taste rating effects. Across exercise conditions, participants rated the control foods as significantly less appealing than the appetitive foods, indicating that the experimental effect on control food consumption cannot be attributed to changes in taste preferences or perceptions.

These results provide evidence of a transfer effect to dietary self-control, but only in terms of indirect compensatory behaviors (i.e., those consumptive behaviors that may satisfy hunger by choosing to consume foods that are perceived as the healthiest alternatives available at the choice point). Direct transfer effects—wherein less appetitive foods are consumed—were not found. While the latter findings are initially counter-intuitive, together these findings suggest that exercise induced enhancements in EF may result in increased choice-related dietary self-control, which in turn moderated the type of food consumed rather than the quantity of food consumed. This explanation aligns well with prior research that has implicated the operation of the DLPFC in dietary self-control. For instance, Hare et al. (2009) reported that in comparison to those with weak self-control, individuals with effective self-control more often made decisions about which foods they would like to eat on the basis of perceived health (e.g., apple) rather than taste (e.g., chocolate bar). Additionally, regardless of individual differences in self-control strength, activity in the ventromedial prefrontal cortex (vmPFC) increased when participants made decisions about which foods to eat, however, increased DLPFC activity was observed only in those participants with effective self-control. Therefore, given that acute bouts of aerobic exercise have been shown to increase blood flow to the PFC, specifically the DLPFC (Yanagisawa et al., 2010), it is possible that choice-related self-control was enhanced following moderate aerobic activity. As a result, those with the largest exercise effects on EF may have chosen to consume foods on the basis of perceived health rather than taste, which explains the moderating effect of EF on the consumption of control foods (i.e., perceived healthier food).

Strengths of this study include the use of several measures of inhibition, and the assessment of a cross-domain transfer to dietary self-control, which has not been previously assessed. Additionally, the use of several different exercise intensity conditions provided a comprehensive assessment of the effects of acute aerobic activity on cognition, and of the cross-domain transfer to dietary self-control. The main limitation of this study was the small sample size, which may have reduced our power to detect some effects. Likewise, the use of an undergraduate sample may have reduced variability in our EF measures and therefore further limited our ability to detect effects. Nonetheless, there is currently a lack of prior research in this area, and therefore the results from this study provide some important preliminary findings.

In summary, our findings provide some support for the contention that a single bout of aerobic activity enhances cognitive function, though the effects were of considerable specificity in terms of measures used, and exercise bout intensity. Some evidence of transfer effects to dietary self-control were also observed, but these were specific to indirect effects (i.e., choice of foods perceived as relatively healthier rather than those perceived as relatively unhealthy). Future research should consider examining the effects of a single bout of aerobic exercise on different facets of EF (e.g., working memory, mental flexibility), to determine if aerobic exercise enhances all facets of cognitive functioning or just inhibition specifically. As well, the inclusion of objectively healthy food options (e.g., fruits and/or vegetables), in addition to the control and appetitive food options, in future studies would help to further elucidate this indirect transfer effect.

### Conflict of interest statement

The authors declare that the research was conducted in the absence of any commercial or financial relationships that could be construed as a potential conflict of interest.

## References

[B1] Aiken L. S. West S. G. (1991). Multiple Regression: Testing and Interpreting Interactions. Thousand Oaks, CA: Sage

[B2] Barella L. A. Etnier J. L. Chang Y. K. (2010). The immediate and delayed effects of an acute bout of exercise on cognitive performance of healthy older adults. J. Aging Phys. Act. 18, 87–98 20181996 10.1123/japa.18.1.87

[B3] Bogg T. Voss M. W. Wood D. Roberts B. W. (2008). A hierarchical investigation of personality and behavior: examining neo-socioanalytic models of health-related outcomes. J. Res. Pers. 42, 183–207 10.1016/j.jrp.2007.05.003

[B4] Chang Y. K. Etnier J. L. (2009). Exploring the dose-response relationship between resistance exercise intensity and cognitive function. J. Sport Exerc. Psychol. 31, 640–656 20016113 10.1123/jsep.31.5.640

[B5] Chang Y. K. Tsai C. L. Hung T. M. So E. C. Chen F. T. Etnier J. L. (2011). Effects of acute exercise on executive function: a study with a tower of london task. J. Sport Exerc. Psychol. 33, 847–865 22262708 10.1123/jsep.33.6.847

[B6] Coles K. Tomporowski P. D. (2008). Effects of acute exercise oFn executive processing, short-term and long-term memory. J. Sports Sci. 26, 333–344 10.1080/02640410701591417 18074301

[B7] Congdon E. Mumford J. A. Cohen J. R. Galvan A. Canli T. Poldrack R. A. (2012). Measurement and reliability of response inhibition. Front. Psychol. 3:37 10.3389/fpsyg.2012.00037 22363308 PMC3283117

[B8] Deighton K. Zahra J. C. Stensel D. J. (2012). Appetite, energy intake and resting metabolic responses to 60 min treadmill running performed in a fasted versus a postprandial state. Appetite 58, 946–954 10.1016/j.appet.2012.02.041 22366285

[B9] Di Renzo L. Rizzo M. Sarlo F. Colica C. Iacopino L. Domino E. (2013). Effects of dark chocolate in a population of normal weight obese women: a pilot study. Eur. Rev. Med. Pharmacol. Sci. 17, 2257–2266 23893195

[B10] Engler M. B. Engler M. M. (2006). The emerging role of Flavonoid-Rich cocoa and chocolate in cardiovascular health and disease. Nutr. Rev. 64, 109–118 10.1111/j.1753-4887.2006.tb00194.x 16572598

[B11] Erdman J. W. Jr. Carson L. Kwik-Uribe C. Evans E. M. Allen R. R. (2008). Effects of cocoa flavanols on risk factors for cardiovascular disease. Asia Pac. J. Clin. Nutr. 17, 284–287 18296357

[B12] Friedman N. P. Miyake A. Young S. E. Defries J. C. Corley R. P. Hewitt J. K. (2008). Individual differences in executive functions are almost entirely genetic in origin. J. Exp. Psychol. Gen. 137, 201–225 10.1037/0096-3445.137.2.201 18473654 PMC2762790

[B13] George V. A. Morganstein A. (2003). Effect of moderate intensity exercise on acute energy intake in normal and overweight females. Appetite 40, 43–46 10.1016/S0195-6663(02)00146-0 12631503

[B14] Guerrieri R. Nederkoorn C. Jansen A. (2012). Disinhibition is easier learned than inhibition. The effects of (dis)inhibition training on food intake. Appetite 59, 96–99 10.1016/j.appet.2012.04.006 22521403

[B15] Guerrieri R. Nederkoorn C. Schrooten M. Martijn C. Jansen A. (2009). Inducing impulsivity leads high and low restrained eaters into overeating, whereas current dieters stick to their diet. Appetite 53, 93–100 10.1016/j.appet.2009.05.013 19467278

[B16] Hall P. A. (2012). Executive control resources and frequency of fatty food consumption: findings from an age-stratified community sample. Health Psychol. 31, 235–241 10.1037/a0025407 21895367

[B17] Hall P. A. Lowe C. Vincent C. (2013). Executive control resources and snack food consumption in the presence of restraining versus facilitating cues. J. Behav. Med. [Epub ahead of print]. 10.1007/s10865-013-9528-3 23943139

[B18] Hare T. A. Camerer C. F. Rangel A. (2009). Self-control in decision-making involves modulation of the vmPFC valuation system. Science 324, 646–648 10.1126/science.1168450 19407204

[B19] Hill A. J. Weaver C. F. Blundell J. E. (1991). Food craving, dietary restraint and mood. Appetite 17, 187–197 10.1016/0195-6663(91)90021-J 1799281

[B20] Hogervorst E. Riedel W. Jeukendrup A. Jolles J. (1996). Cognitive performance after strenuous physical exercise. Percept. Mot. Skills 83, 479–488 10.2466/pms.1996.83.2.479 8902021

[B21] Houben K. Jansen A. (2011). Training inhibitory control. A recipe for resisting sweet temptations. Appetite 56, 345–349 10.1016/j.appet.2010.12.017 21185896

[B22] IBM Corp. (2012). IBM SPSS Statistics for Windows, Version 21.0. Armonk, NY: IBM Corp

[B23] King N. A. Burley V. J. Blundell J. E. (1994). Exercise-induced suppression of appetite: effects on food intake and implications for energy balance. Eur. J. Clin. Nutr. 48, 715–724 7835326

[B24] King N. A. Lluch A. Stubbs R. J. Blundell J. E. (1997a). High dose exercise does not increase hunger or energy intake in free living males. Eur. J. Clin. Nutr. 51, 478–483 10.1038/sj.ejcn.1600432 9234032

[B25] King N. A. Tremblay A. Blundell J. E. (1997b). Effects of exercise on appetite control: Implications for energy balance. Med. Sci. Sports Exerc. 29, 1076–1089 10.1097/00005768-199708000-00014 9268966

[B26] Kuntsi J. Andreou P. Ma J. Borger N. A. van der Meere J. J. (2005). Testing assumptions for endophenotype studies in ADHD: reliability and validity of tasks in a general population sample. BMC Psychiatry 5:40 10.1186/1471-244X-5-40 16262903 PMC1291376

[B27] Lambourne K. Tomporowski P. (2010). The effect of exercise-induced arousal on cognitive task performance: a meta-regression analysis. Brain Res. 1341, 12–24 10.1016/j.brainres.2010.03.091 20381468

[B28] Logan G. D. Cowan W. B. Davis K. A. (1984). On the ability to inhibit simple and choice reaction time responses: a model and a method. J. Exp. Psychol. Hum. Percept. Perform. 10, 276–291 10.1037/0096-1523.10.2.276 6232345

[B29] Miyake A. Friedman N. P. Emerson M. J. Witzki A. H. Howerter A. Wager T. D. (2000). The unity and diversity of executive functions and their contributions to complex “frontal lobe” tasks: a latent variable analysis. Cogn. Psychol. 41, 49–100 10.1006/cogp.1999.0734 10945922

[B30] Nederkoorn C. Guerrieri R. Havermans R. C. Roefs A. Jansen A. (2009). The interactive effect of hunger and impulsivity on food intake and purchase in a virtual supermarket. Int. J. Obes. 33, 905–912 10.1038/ijo.2009.98 19546869

[B31] Pontifex M. B. Hillman C. H. Fernhall B. Thompson K. M. Valentini T. A. (2009). The effect of acute aerobic and resistance exercise on working memory. Med. Sci. Sports Exerc. 41, 927–934 10.1249/MSS.0b013e3181907d69 19276839

[B32] Ried K. Sullivan T. Fakler P. Frank O. R. Stocks N. P. (2010). Does chocolate reduce blood pressure? A meta-analysis. BMC Med. 8:39 10.1186/1741-7015-8-39 20584271 PMC2908554

[B33] Rotenberg K. J. Lancaster C. Marsden J. Pryce S. Williams J. Lattimore P. (2005). Effects of priming thoughts about control on anxiety and food intake as moderated by dietary restraint. Appetite 44, 235–241 10.1016/j.appet.2004.09.001 15808897

[B34] Sibley B. A. Etnier J. L. Le Masurier G. C. (2006). Effects of an acute bout of exercise on cognitive aspects of stroop performance. J. Sport Exerc. Psychol. 28, 285–299 20181996

[B35] Strauss G. P. Allen D. N. Jorgensen M. L. Cramer S. L. (2005). Test-retest reliability of standard and emotional stroop tasks: an investigation of color-word and picture-word versions. Assessment 12, 330–337 10.1177/1073191105276375 16123253

[B36] Stroop J. R. (1992). Studies of interference in serial verbal reactions. J. Exp. Psychol. Gen. 121, 15–23 10.1037/0096-3445.121.1.15

[B37] Taubert D. Berkels R. Roesen R. Klaus W. (2003). Chocolate and blood pressure in elderly individuals with isolated systolic hypertension. JAMA 290, 1029–1030 10.1001/jama.290.8.1029 12941673

[B38] Tomporowski P. D. Ganio M. S. (2006). Short-term effects of aerobic exercise on executive processing, memory, and emotional reactivity. Int. J. Sport Exerc. Psychol. 4, 57–72 10.1080/1612197X.2006.9671784

[B39] Yanagisawa H. Dan I. Tsuzuki D. Kato M. Okamoto M. Kyutoku Y. (2010). Acute moderate exercise elicits increased dorsolateral prefrontal activation and improves cognitive performance with stroop test. Neuroimage 50, 1702–1710 10.1016/j.neuroimage.2009.12.023 20006719

